# Scalp hair sweating as a predictor of hair cortisol level in human compared to obesity and other confounders

**DOI:** 10.1038/s41598-021-02223-0

**Published:** 2021-12-17

**Authors:** Darya S. Abdulateef

**Affiliations:** Physiology Department, College of Medicine, University of Sulaimani, New-Street-27, Zone 209, P.O. Box: 334, Sulaymaniyah, Kurdistan Region Iraq

**Keywords:** Biomarkers, Medical research

## Abstract

Inconsistent results were found throughout the literature regarding factors affecting hair cortisol levels. Hair cortisol level in humans was not studied for its associations to scalp hair sweating or hair wash frequency in a patient-based way. Factors affecting hair cortisol levels must be precisely known in order to interpret the results correctly. The aims of the study are to assess if BMI, Perceived Stress Scale (PSS), hair wash frequency, and sweating with scalp hair affect hair cortisol levels. It will assess which of these factors are more significant predictors of hair cortisol levels. In a study on healthy adults, information about history, socio-demographics, PSS, hair wash frequency, hair treatment, and scalp hair sweating were collected, and hair samples were taken and analyzed for their hair cortisol level. Associations of hair cortisol levels with each of the variables were investigated and significant predictors of hair cortisol levels among the variables were found. Mean hair cortisol level in the study participants was 16.84 pg/mg hair. Hair cortisol has a significant positive association with weight, BMI, PSS, and scalp hair sweating, p < 0.05. Scalp hair sweating significantly predicts hair cortisol levels by 12.3%, while other variables did not significantly predict hair cortisol levels, p < 0.05. Scalp hair sweating significantly predicts hair cortisol levels. Age, hair wash frequency, hair treatment, and stressful events have no associations with hair cortisol levels. Although BMI and PSS are associated with hair cortisol levels, they do not significantly predict it. Obesity is significantly associated with profuse sweating, thus the increase in hair cortisol levels in obese individuals could partly be the result of a higher incidence of sweating in these individuals. Thus, scalp hair sweating should be taken into consideration during the study and interpretation of hair cortisol levels.

## Introduction

One of the common health problems in the world, nowadays, that suffered by many adults^1^ and adolescents is stress^1^. Thus, recently, there has been increased attention in assessing the level of stress and measuring stress in a subjective way, via questionnaires^2^.

Analyzing stress by using subjective measure alone is insufficient; it is better to be accompanied by objective measures^2^. The subjective measure of stress could be greatly affected by some subjective matters or personal experiences of the individuals. The biomarker that used commonly to assess the stress objectively by finding the physiological level of stress and agreed on worldwide is cortisol level^2^.

Previously, researchers measured cortisol level in blood, saliva, or urine. Measuring of cortisol level in these samples can be used to assess its level only acutely, or over a short period of time, further, it is affected by diurnal variations. While investigating cortisol level in the hair can be used to assess the level of stress chronically, and it is indicative of the activity of hypothalamic pituitary adrenal (HPA) axis^3–5^ and is a valid test that has been used in various researches on the animal as well as human subjects^3–14^. Being a fat soluble substance, free cortisol integrated into the matrix of the hair^15^, and as the average monthly hair growth is about 10 mm, it can be used as a retrospective estimate of cortisol levels over several months with a one-time sample collection^16^. Thus, a 3 cm length of hair sample represents the cortisol exposure of the three preceding months. Several hormones were detected in the human hair samples throughout the studies, including reproductive hormones, thyroid hormones, 25-hydroxy vitamin D, and C-peptide^17–21^.

Collection of the scalp hair is an easy procedure and can be performed in outpatient^22^. The hair sample can easily be stored with no need for a special storage procedure; it remains stable for several months simply by putting it into a foil in a dark, dry place at room temperature^4,23–25^.

Cortisol in normal subjects was measured in several studies at an average level of about 5.9–22.6 pg/mg hair. In studies of adults more than 50 years old, its level was 21–40.5 pg/mg hair^26^. Studies also found higher cortisol levels in obese subjects compared to normal weighted subjects^27–29^. Apart from age and BMI, several other factors could be regarded as confounders for hair cortisol level, among them hair treatment by bleaching, hair shampooing, and frequency of hair washing with or without hot water^30–32^. Sweating is another factor that could affect hair cortisol level. An increased amount of cortisol in sweat was found by researchers after acute stress^33^ and higher hair cortisol level was found in endurance athletes^34^ and those with higher levels of physical activity^35,36^. In these individuals, sweating was discussed to have a role in their higher hair cortisol level, while the pattern of sweating and the effect of scalp hair sweating on hair cortisol levels was not assessed in these studies. More recently, sweating was found to be one of the confounder of hair cortisol level, as questioned from participants in a study investigating different confounders among them head sweating^9^. Studies on the relationship between the objective (hair cortisol) and the subjective (PSS) indicators of stress were inconsistent throughout literature^32^. The exact relation of hair cortisol to PSS was not known, neither was the exact relation of hair cortisol to hair washing frequency or sweating patterns.

## Aims of the study

To find significant predictors of hair cortisol level among socio-demographic and other confounders, such as PSS, hair wash frequency, use of hair products (gel or cream), and scalp hair sweating, with the focus on two important related parameters; BMI and sweating, to investigate which one of them is a better predictor of hair cortisol level. This is important, as subjects with a high BMI are usually associated with profuse sweating. We hypothesized that the pattern of scalp hair sweating will affect hair cortisol level after correction for BMI and other confounders.

## Method

In a prospective study at General teaching hospital, Sulaymaniyah city, 127 individuals have participated in the study. The study includes healthy individuals with age between 18–70 years, who were either the hospital staff, relatives, or patient accompanies. Exclusion criteria include those with; chronic diseases, acute illnesses, HPA axis abnormality, on treatment for thyroid disease, on treatment and medications affecting HPA axis, inpatients, pregnant and those who had a psychological problem. Written informed consent was taken from all participants. The proposal with all experimental protocols was accepted by the Ethical Committee, College of Medicine University of Sulaimani, under the meeting no. 10, on August 16th, 2020, and all experiments were performed in accordance with their guidelines and regulations.

A questionnaire with history and socio-demographic status was filled for each participant, including a detailed query about frequency of hair wash, use of gel or cream, history of major stressful events within the three months (for example death of the first degree relatives or beloved one, losing a job or witness to war, explosion, or a road traffic accident and etc.) and a detailed questionnaire about the sweating pattern and scalp hair sweating. The participants were questioned for Perceived Stress Scale (PSS) points (Supplementary material 1), the scale was filled, and the sums of the score were reported for each participant.

The sweating questionnaire (Supplementary material 2) includes a general question about sweating, if the person sweats or not choosing one of the four choices provided according to the daily activity and environmental temperature. In the next question, the individuals specify the body part that the person sweats more than the other. Then more specific questions were asked to determine if they sweat with their scalp or not, and if the answer is yes, which part of the scalp do they sweat, anterior or posterior part. The individuals were indicating the pattern of their sweating to choose between the four answers, whether during the sweating, their hair humid, wet, soaked and dripping or soaked and very dripping. Then the amount of sweating was determined according to the questions about the situation they sweat and the sweating pattern, into four different groups, no, little, moderate or profuse sweating.

### Hair sample taking, preparation, extraction, and analysis

Scalp hair was taken from the posterior vortex. About a half cm thickness of hair was strapped and cut above the strapped area with clean scissors at the most proximal point to the scalp. The proximal three cm of the cut hair was then conserved in aluminum foil, kept in a paper envelop and stored in a dark, dry place at room temperature until the time of analysis.

### Exclusion criteria for hair sample taking include dying hair or insufficient hair

Before analysis, the hair samples were washed two times first incubated at room temperature with 2.5 ml of isopropanol for 2 min and the isopropranolol were discarded and then washed with distilled water using the same amount and duration. After drying, the hair samples were cut with surgical scissors into a two to three mm length and weighed with an electronic analytical balance. About 20–50 mg of each hair sample was put into a glass test tube and 1.5 ml of methanol was added with a calibrated micro-pipette. Then these test tubes were put into a shaking incubator at 52 °C and left for 16 h. After incubation, the test tubes were centrifuged at 5000 rpm for 10 min, then the supernatant (the methanol with its extract) was separated into other glass test tubes. These test tubes were then put on a heating block until drying. After the process of drying, about 0.2 ml of phosphate buffer saline (PBS) was added to the precipitate, and they were vortexed for 30 s two times in order to mix them well. The samples were then analyzed by an Electrochemiluminescence assay (ECLA) Roche Diagnostic COBAS e-411, with Roche diagnostic c-peptide kit. Later, the amounts of cortisol within each mg of hair were calculated in pg/mg.

The subjects were divided into groups of overweight/obese or normal weighted subjects according to their BMI. Normal weight: any subject with BMI between 18.5 and 24.9 kg/m^2^. Under weight: any subject with BMI below 18.5 kg/m^2^. Overweight and obese: any subject with BMI equal or above 25 kg/m^2^ and based on their cortisol level; they were divided into higher or lower cortisol level group, according to the mean cortisol level of 20.86 pg/mg as the cut-off value.

### Statistical analysis

The data were analyzed using SPSS, the values were investigated for normality distribution using the Kolmogorov normality test, and the mean and standard deviations were presented for parametric values, while non-parametric values were presented as median and range (IQR). Hair cortisol, age, and frequency of hair wash were log transformed for correction of skew. Correlation of hair cortisol with PSS, frequency of hair wash, degree of sweating, and other variables were assessed using Pearson’s correlations, and significant correlations were shown using scatterplot matrices. Comparisons between groups according to BMI and amount of sweating were done using Student *t* test and ANOVA, and their data were presented via bar charts, with the significant level set at 0.05 (p value ≤ 0.05). The significant predictors of hair cortisol level were investigated among available parameters by using Multiple Linear Regression analysis, and because of collinearity between variables, Step-wise Multiple regressions were used and p value ≤ 0.05 was regarded as a significant predictor. Bonferroni correction was made for multiple linear regression analysis.

## Results

Among the 127 individuals who participated in the study, 102 subjects were eligible and their parameters were assessed. After the exclusion of 32 subjects with either extreme hair cortisol levels, deviation of more than 4 SD of the mean, incomplete filling of the PSS questionnaire, or other missing data, 70 subjects remained for the final study analysis.

The median age of the study participants was 32.5 (range: 18–65) years, with a median hair cortisol level of 16.84 (range: 3.59–69.12) pg/mg hair and a mean PSS level of 18.97. 57% of the participants responded that they had no scalp hair sweating, and among those with scalp sweating, only 8% had profuse sweating. The results of socio-demographic and other parameters were demonstrated in Table 1.

**Table 1:** Sociodemographics and general characteristics of the studied participants.

Parameters	Mean (SD) or Median [IQR] Frequency (%)	No
**Age (years)**	32.5 [IQR: 17, 24–40.75]	70
Range	18–67	
**Gender**		
Male	8 (11.4%)	70
Female	62 (88.6%)	
**BMI (kg/m**^**2**^**)**	26.65 (5.24)	70
Range	17.17–41.74	
**BMI groups**		70
Normal weight	28 (40%)	
Overweight and obese	38 (54.3%)	
Underweight	4 (5.7%)	
**Waist circumference (cm)**	83.74 (10.96)	52
Range	64–105	
**Median hair cortisol (pg/mg hair)**	16.84 [IQR: 17.67, 9.03–26.7]	70
Range	3.59–69.12	
Higher hair cortisol	30.34	
Lower hair cortisol	10.25	
Log hair cortisol	1.221 (0.293)	70
**Hair cortisol groups**		70
Higher hair cortisol	26 (37.1%)	
Lower hair cortisol	44 (62.9%)	
Hair wash frequency (times/week)	3 [IQR: 1.5, 2–3]	69
Range	0.5–7	
**Sweating with scalp hair**		70
No	39 (55.7%)	
Yes	31 (44.3)	
Little	20 (28.6%)	
Moderate	5 (7.1%)	
Profuse	6 (8.6%)	
Sweating with scalp hair	1.69 (0.94)	70
Gel/Cream after wash	9 (13.6%)	66
Stressful event	23 (34.3%)	67
**PSS**	18.97 (5.45)	70
Range	6–30	

The descriptive value presented as mean (SD) or median [interquartile range IQR] and ranges for non-parametric values. The numeric variables were shown as frequency (%).

**Figure 1:**
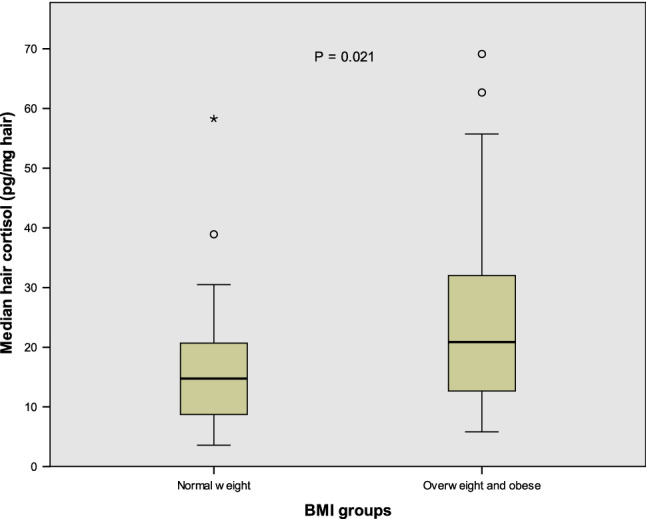
Hair cortisol level in overweight/obese subjects compared to normal weighted subjects.

Median hair cortisol levels in the overweight and obese were significantly higher in comparison to normal persons (20.86 vs 14.76 pg/mg hair) as shown in Fig. 1, p = 0.021. Hair cortisol in underweight was lower than normal weight subject with the median of 11.36 pg/mg hair, but the small number of participants in the underweight group (N = 4) did not allow correct statistical comparison with other groups.

**Figure 2:**
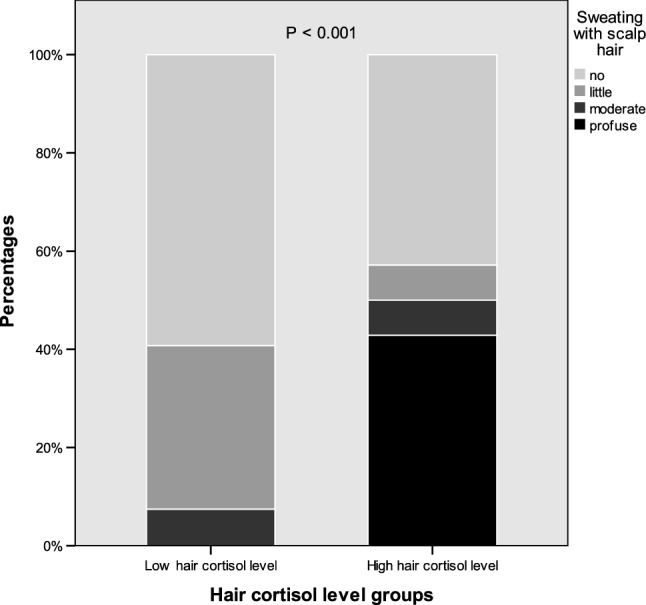
The amount of sweating in percentages between subjects with lower and higher hair cortisol levels.

All subjects with profuse sweating were among the higher hair cortisol group, and 42.9% of the group with higher cortisol has profuse sweating, compared to 0% of profuse sweating among subjects with lower cortisol level, p < 0.001, Fig. 2. At the same time, 92.3% of the subjects within the lower hair cortisol group have either no sweating (59%) or mild sweating (33.3%) with scalp hair.

**Figure 3:**
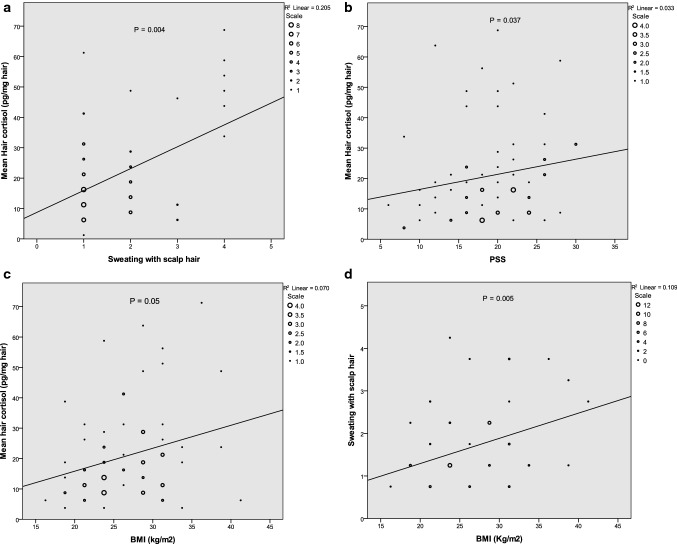
Scatterplot correlations between hair cortisol level and scalp hair sweating with other variables. Notes: the scales (circular areas) indicate the count of records to visualize the data point density.

Figure 3 shows the scatter plot of correlation between hair cortisol level and scalp sweating, BMI, and PSS, which revealed a significant positive association (r = 0.337, p = 0.004; r = 0.235, p = 0.05 and r = 0.249, p = 0.037, respectively) and a positive correlation between scalp hair sweating and BMI, r = 0.330, p = 0.005. Other correlations showed significant positive association between scalp sweating and age, r = 0.269, p = 0.024) and, scalp sweating with waist circumference, r = 0.438, p = 0.001).

Hair cortisol level has no significant association with age and gender, as well as no significant correlation with hair wash frequency, gel or cream use, or history of stressful events (r = 0.039, p = 0.747; r = − 0.013, p = 0.916 and r = 0.030, p = 810) respectively.

Multiple linear regression analysis revealed that none of the parameters apart from scalp hair sweating significantly predict hair cortisol level. Scalp hair sweating predicts hair cortisol level by 12.3%, p = 0.004, beta coefficient of 0.108; it remains significant after Bonferroni correction with a new p-value of 0.007 and because the dependent variable; hair cortisol is log transformed, Supplementary material 3.

## Discussion

The association of elevated hair cortisol level with stress scales like PSS made researchers use PSS as a common stress scale, in addition to the use of the biological measure of chronic stress. PSS is a self-reported questionnaire developed by Cohen et al.^37^. Significant correlation between hair cortisol and PSS was found in the current study, although after correcting for other confounders, PSS was not a significant predictor of hair cortisol levels. Inconsistent results regarding hair cortisol and PSS were found by researchers. Positive associations between PSS and hair cortisol level were found in a cross-sectional study on middle-aged women^38^, while negative associations were found in a collected database of community samples. The researcher suggested that the intensity of the stressor might have a great effect on the level of hair cortisol^39^. Several other studies could not find an association of elevated hair cortisol levels with PSS^2,40–43^, for instance in women living in a socioeconomically disadvantaged neighborhood in the READI study^40^, in a study among a group of pregnant women^41^, in a study on healthy adolescents^2^, in study on women in one of the poorest cities in the United States^42^ and in a meta-analysis, to investigate the basic determinant of hair cortisol level by Stalder et al.^32^. At the same time, Weckesser et al. found no association between hair cortisol and PSS, while they found a significant association with the weekly Hussle scale (once per week)^43^.

Controversy exists on the relation of age and gender with hair cortisol level. In line with most previous studies, the present study found no differences in hair cortisol levels in regards to the age^26,44^ or gender^12,44–46^ of the studied participants, while in a few other studies, inconsistently higher cortisol levels with increasing age^6,9,47^ and lower hair cortisol levels in female in comparison to male participants^6,9,26^ were found. Differences in sample size, the age range studied, and the technique of hair preparation and analysis could be the cause of these discrepancies, although regarding gender, the small number of males in our study could have led to an inadequate analysis of gender differences.

This study supports several other studies^9,12,22,28,29,48,49^ where hair cortisol was related to BMI, and found a significant positive correlation between BMI and the hair cortisol level of the studied participant. Although in the current study, consistent with other studies^27,40,50^, overweight and obese participants had a higher hair cortisol level compared to normal, when other cofounders were removed, BMI did not regarded as a significant predictor of hair cortisol levels. We also could not find a significant correlation between hair cortisol and waist circumferences. Similarly, a significant correlation between hair cortisol level and BMI and waist circumference was not found in a study done in Amsterdam on elderly individuals^26^. In a study on 300 adults and children, no differences in hair cortisol levels were found between subjects of a normal weight and the overweight and obese^51^.

Hair cortisol level is affected by hair treatment products^14^, and it may be affected by sweating, natural hair color and type^14^, frequency of hair washing^32,48^, and some environmental factors. Thus, considering these factors during hair cortisol assessment is important to prevent falsely low or high cortisol levels due to hair dyeing or excessive sweating^52^.

In a meta-analysis by Stalder et al. on 66 independent studies, hair wash frequency were found to be among covariate to be considered during hair cortisol assessments. In this study, hair washing frequency and use of hair products, such as gel or cream, have no significant association with hair cortisol levels. In line with the present study, most of the studies on human hair did not show a significant association between hair cortisol levels and hair washing frequency^6,9,12,48^ except for the most distal part of the hair; the third segment^6^, which is of less concern, because measuring the proximal three cm of hair (or six cm if required) is a typical procedure that can give enough data on an individual’s condition. While conducting an experimental study on Rhesus monkeys^31^, researchers revealed that hair cortisol levels are affected by repeated hair washing and are negatively associated with frequency of hair washing with or without use of shampoo. In this study, they did not specify the length of hair used, and they may have used the more distal hair samples from the scalp. This would explain this discrepancy, as distal samples are associated with a lower hair cortisol level, and they are associated with a greater decrease in hair cortisol by hair washing. Furthermore, the factors that affect hair cortisol could be different in animals compared to humans.

Studies found that hair treatments such as hair dyeing affect hair cortisol levels significantly and could give false low results^12,14^. In this study, we excluded individuals with dyed hair to remove the effect of this confounder.

In a study on young, healthy adults, individuals with vigorous physical activity were associated positively with high cortisol levels, while this association was not seen in subjects of moderate physical activity. This suggests that the higher stressors in subjects with vigorous physical activity could be the causative factor for higher hair cortisol levels^36^. Also, high cortisol levels were found in elite athletes, with excessive sweating proposed to be the causative factor^3,34,35^.

The researchers proposed the likelihood of higher hair cortisol levels in the hair of individuals with a greater amount and duration of sweating at the time of sample taking. The washing of the sample during preparation was of no help^47^. In an experimental human study to induce sweating, the participants were engaged in physical exercise or sauna bathing to induce sweating, the researchers suggested that hair cortisol levels will not be acutely affected by activity associated with sweating at the time of procedure^53^*.* Another study was conducted on endurance athletes, found higher hair cortisol levels in the endurance athletes, and they suggested that intense, repeated exercise has an effect on hair cortisol in a prolonged, non-acute way^34^. This finding, together with the absence of elevated PSS in subjects with elevated hair cortisol reached through vigorous physical activity^36^, could be suggestive of a general sweating pattern. Repeated excessive sweating in these individuals could be the causative factor in the elevation of hair cortisol levels.

In more recent study about hair cortisol assessment, Enge et al.^9^, in a questionnaire about sociodemographic and hair characteristics of participants, they included a question about head sweating, whether they were aware of it or not. Inline with the current study, Enge et al. study revealed a significantly positive association between hair cortisol level and scalp sweating, but inconsistent with our study, BMI besides scalp sweating remain as a strong covariate influencing hair cortisol level^9^. Although the number of the study sample was greater in Enge et al. study compared to the present study, most of their participants were among the self-collector of hair samples for hair cortisol measurement and BMI were recorded via questionnaire, rather than direct measurement of height and weight by the medical staff during conducting the research, these could have an effect on the hair cortisol and BMI level and on the differences between the two study results. Furthermore, the main study aims of Enge et al. were different from the current study, and thus the question asked about scalp sweating was a simple question and answered by choosing one of the answer among three choices without going to the detail.

High BMI is another factor that is associated with excessive sweating^54^, thus the increase in hair cortisol levels in the overweight and obese could partly be due to higher sweating in these individuals in comparison to those of normal weight, rather than the direct effect of BMI on hair cortisol levels. The presence of scalp hair sweating as the only predictor of high hair cortisol levels in normal healthy people with normal PSS after removal of other confounding factors in the present study will support this idea. Profuse sweating is associated with higher hair cortisol levels in comparison to mild to moderate sweating. We could suggest that sweating would have a role in the elevation of hair cortisol levels even after the washing of the hair, thus the elevation might be the result of the incorporation of sweat into the inside of the hair in cases with excessive sweating on a usual basis, rather than just covering the hair superficially by acute sweating.

The limitations of this study include; small sample size, unequal gender distribution due to insufficient hair in the posterior vertex region of the scalp in a group of male participants due to balding or the hair cut style, and failure to exclude smokers, lactating women, or drug use. Another shortcoming and challenge that we faced is the use of subjective measurement of scalp hair sweating. The use of actual sweat collections to investigate the amount of sweating could be associated with more accurate and unbiased results compared to subjective measure. Unfortunately, sweat amount couldn’t be measured objectively because of the difficulty of getting or quantifying the sweat amount on the scalp hair, like what was done for other region of the body via using wearable sweat rate sensor device^55^; especially on the posterior vertex covered with the hair, the portable instrument could not be placed in this region. Failure to interpret the multiple regression analysis data in detail is another limitation of the current study, because log transformed hair cortisol level used and the mean cortisol level were used as the cut-off value of high and low cortisol level.

Moreover, the use of author designed questionnaire which was not validated with the actual sweat amount is regarded as another study limitation.

In conclusion, scalp hair sweating is the significant predictor of hair cortisol level. BMI, PSS, hair wash frequency and other variables could not significantly predict hair cortisol level when controlling for other confounders. We rejected the null hypothesis that scalp hair sweating could not significantly predict hair cortisol level. It is important to record the subject’s history of sweating before taking the hair sample for hair cortisol analysis, and individuals associated with profuse sweating patterns should be recognized. We recommend future studies on scalp sweating and hair cortisol level association among a large group of obese and non-obese individuals, trying to find a new technique to quantify the scalp sweating, and use the objective measure of scalp hair sweating to predict the hair cortisol level for further evaluation and validation of this finding. We also especially recommend focusing on the subjects’ history of sweating and sweating patterns during hair cortisol investigation, especially in subjects with a high BMI.

## Supplementary Information

Supplementary Information.

## Data Availability

All research data available under Figshare repository file under the DOI of 10.6084/m9.figshare.14414489.
