# Supplementary material for: Scalp hair sweating as a predictor of hair cortisol level in human compared to obesity and other confounders
Source: Sci Rep. 2021 Dec 17;11:24160. doi: 10.1038/s41598-021-02223-0 (PMC8683402; doi:10.1038/s41598-021-02223-0)
Supplement: Supplementary file 1 — Supplementary Information. [file 41598_2021_2223_MOESM1_ESM.pdf]

Supplementary material 1. Perceived Stress Scale

The questions in this scale ask you about your feelings and thoughts during the last month**. In each case, you will be asked to indicate by circling *how often* you felt or thought a certain way.**

Name____Date _____________

Age_______Gender (*Circle*): **M F** Other ______________________________

0 = Never 1 = Almost Never 2 = Sometimes 3 = Fairly Often 4 = Very Often

1. In the last month, how often have you been upset because of something that happened unexpectedly?	0	1	2	3	4
2. In the last month, how often have you felt that you were unable to control the important things in your life?	0	1	2	3	4
3. In the last month, how often have you felt nervous and “stressed”?	0	1	2	3	4
4. In the last month, how often have you felt confident about your ability to handle your personal problems?	0	1	2	3	4
5. In the last month, how often have you felt that things were going your way?	0	1	2	3	4
6. In the last month, how often have you found that you could not cope with all the things that you had to do?	0	1	2	3	4
7. In the last month, how often have you been able to control irritations in your life?	0	1	2	3	4
8. In the last month, how often have you felt that you were on top of things?	0	1	2	3	4
9. In the last month, how often have you been angered because of things that were outside of your control?	0	1	2	3	4
10. In the last month, how often have you felt difficulties were piling up so high that you could not overcome them?	0	1	2	3	4

mind garden

info@mindgarden.com
www.mindgarden.com

References

The PSS Scale is reprinted with permission of the American Sociological Association, from Cohen, S., Kamarck, T., and Mermelstein, R. (1983). A global measure of perceived stress. *Journal of Health and Social Behavior, 24,* 386-396.
Cohen, S. and Williamson, G. Perceived Stress in a Probability Sample of the United States. Spacapan, S. and Oskamp, S. (Eds.) *The Social Psychology of Health.* Newbury Park, CA: Sage, 1988.

Supplementary material 2.
Hair wash and Scalp hair sweating questionnaire

Code: ………….

Part I

A. Are you exercising daily?

1. No 2. Yes , Duration of daily exercise: ……. min

B. How many times you wash your hair per week? ……………….. Times/week

C. Are you using cream, gel, wax or spray after wash?

1. No 2. Yes

D. Do you have history of major stressful event within last three month?

1. No 2. Yes

Part II
Sweating questionnaire

E. Do you have sweating
   1. No or rarely
   2. Only on a hot day or only during exercise
   3. During usual daily activity in a temperate temperature
   4. During rest or in a cold day

F. Which part of your body sweat more (major area of your sweating)?

Armpit   Back or chest   Hands or feet   Head

G. Do you have sweating with your scalp head?

1.No   2.Yes

If yes, which part of scalp do you sweat more?

1. Near forehead   2. Posterior scalp (Vertex)

H. How do you describe your head sweating (your scalp hair)?
1. Humid   2. Wet   3. Soaked and dripping   4. Soaked and very dripping

---

According to E, G and H the amount of sweating is one of the following
1. No (E1 and G1)
2. Little (E2 and H1 or H2)
3. Moderate (E3 and H2 or H3)
4. Profuse (E4 and H4, or E3 and H4, or E4 and H3)

Supplementary material 3. Sociodemoghraphic, psychosocial and hair related parameters to predict hair cortisol level through multiple linear regressions.

Model Summary[b]

Model	R	R Square	Adjusted R Square	Std. Error of the Estimate	Change Statistics					Durbin-Watson
					R Square Change	F Change	df1	df2	Sig. F Change	
1	.351[a]	.123	.109	.28223	.123	8.706	1	62	.004	1.755

a. Predictors: (Constant), SweatingWithScalpHair

b. Dependent Variable: LogCortisolAll

Coefficients[a]

Model		Unstandardized Coefficients		Standardized Coefficients	t	Sig.	95.0% Confidence Interval for B	
		B	Std. Error	Beta			Lower Bound	Upper Bound
1	(Constant)	1.039	.071		14.636	.000	.897	1.181
	SweatingWithScalpHair	.108	.037	.351	2.951	.004	.035	.181

a. Dependent Variable: LogCortisolAll

Excluded Variables[a]

Model		Beta In	t	Sig.	Partial Correlation	Collinearity Statistics
						Tolerance
1	LogAge	.161[b]	1.311	.195	.165	.922
	BMI	.161[b]	1.294	.201	.163	.902
	PSS	.176[b]	1.479	.144	.186	.982
	LogHairwash	.043[b]	.354	.725	.045	.984
	GelCremYesNo	.052[b]	.428	.670	.055	.962
	StressfulEvent3monthsAgo	.026[b]	.217	.829	.028	.999

a. Dependent Variable: LogCortisolAll

b. Predictors in the Model: (Constant), SweatingWithScalpHair

Note: beta coefficient of 0.108, while the dependent variable; hair cortisol is log transformed, thus the exponentiation of the coefficient with subtracting from one and multiplying by 100,

[Exp (0.108)-1] X 100 → 11.4

thus with every one unit increase in sweating there will be an increase of hair cortisol level by 11.4%
